# Dental Materia Medica

**Published:** 1866-10

**Authors:** Geo. Watt


DENTAL REGISTER.

VOL. XX.] OCTOBER, 1866. [No. 10.

### Original Communications.

### DENTAL MATERIA MEDICA.

BY GEO. WATT.

### SUBPHOSPHATE OF LIME.

LIME and phosphoric acid form more than one combination. Three equivalents of the acid united with four of the base constitute the salt under consideration. It was known to be a constituent of bones as early as 1769. To distinguish it from other phosphates of lime, and especially from the neutral, it is called *subphosphate*, or *bone-phosphate.*

This salt is the principal earthy ingredient of the bones of the vertebrate animals and of the shells of the crustaceous.

PREPARATION.—When bones are burned in open vessels, the animal matter is driven off, and what is known as the bone-ash remains. This is composed mainly of the subphosphate and carbonate of lime, the former greatly preponderating. The subphosphate often plays the part of a base—that is, it unites directly with acids, without undergoing decomposition. Not so with the carbonate, which is very readily decomposed. Hence, if bone-ash is digested with hydrochloric acid, the carbonate is decomposed, its carbonic acid being set free, while the chloride of calcium and water are formed, as indicated by the following equation:

$$CaO, CO_2 + HCL = CaCl + HO + CO_2 .$$

Chloride of calcium, being highly soluble, remains in solution. And, as the acid forms a soluble compound with the subphosphate, the result of the digestion is a complete solution of the bone-ash. Now, to precipitate the subphosphate, it is only necessary to give the acid something it likes better; and, as alkalies are the favorites with acids, ammonia may be used, and added to the solution as long as a white powder is precipitated. This powder is the subphosphate of lime, and may be washed with clean water, to remove traces of other salts.

This general direction would enable any one having the slightest acquaintance with chemistry to prepare the salt for himself; but as some of my readers may be more pharmaceutical than chemical, it may be well to give a definite formula:

Take of bones, burnt and pulverized, 1 part;
Hydrochloric acid, 2 parts;
Water, 2 parts.

Mix, and let stand twelve hours, and then filter the liquor. Then add aqua ammonia as long as a precipitate falls. Wash and dry the precipitate, which is the salt under consideration.

If any dentist sees fit to prepare the salt for himself, he will find the largest sized Hessian crucibles and his melting furnace all that he can desire for calcining the bones; and all else that is needed may be found in almost any dental establishment.

Properties.—This salt is a white, tasteless, inodorous, gritty powder, insoluble in water, but readily soluble in hydrochloric, acetic, lactic, and nitric acids. From its solutions in these acids, it is thrown down, unchanged, by solutions of the alkalies, or their carbonates. It melts at a very high heat, without decomposition or other change. It is surely not accidental that the basis of the solid framework of our bodies is composed of this *permanent*, almost indestructible salt.

PHYSIOLOGICAL EFFECTS.—If I were simply to follow the authority of writers on materia medica, it would be enough to say, "Its effects are not very obvious;" and this or similar remarks are so frequent, that I am led to believe that authors have mostly copied each other, instead of giving their individual views as a result of experiment and observation. Nor does it appear to me that they bear in mind its chemical properties, even when they state them. For example, they object to its efficiency as a medicine, that it is nearly insoluble. Insoluble in what? Have they not just told us that it is highly soluble in nitric, hydrochloric, acetic, and lactic acids, and slightly so in solutions of chloride of sodium and carbonic acid? And, as a majority of these are at home in the human stomach, and none of them need be strangers there, what becomes of their objection?

That this salt is an important constituent in the bones, all know; but too many look upon the bony system as a matter of course, and, at the same time, fail to recognize the parts played by the phosphate in other tissues of the system. Its importance to the constitution may be inferred from a few observations.

Lehman (vol. 1, p. 374,) says: "We observe that softening of the bones occurs in those conditions when the animal organism does not receive a sufficient amount of phosphate of lime, or when certain physiological processes require an increased consumption of this salt, as in pregnancy, and during the dentition of children. We need hardly remark that rachitis frequently, if not always, occurs simultaneously with the period of dentition, that the consumption of phosphate of lime during pregnancy is often so great that scarcely any traces of it can be found in the urine; and that, during this period of woman's life, fractures unite with extreme difficulty, and sometimes do not unite at all. Chassat was able to induce softening of the bones artificially in animals, when he restricted them to food containing little or no phosphate of lime."

Again he says (page 375): "Phosphate of lime also occurs in many other parts of the animal body, although in far less quantity than in the bones; indeed, there is no animal tissue in whose ash or incineration we do not find phosphate of lime." On the same page he says: "Phosphate of lime is found in solution in all the animal fluids." *In solution!* Then it is dissolved by the system in some way.

The importance of this salt in the animal economy will farther appear from another quotation from the same author (page 376): "The constant occurrence of this salt in the histogenetic substances, and especially in the plastic fluids, as well as its deposition in many pathologically degenerated cells of the animal body, obviously strengthen the opinion that this substance plays an important part in the metamorphosis of the animal tissues, and especially in the formation and in the subsequent changes of animal cells."

In speaking of the phosphate, Wibmer says it "increases, incontestably, the presence of calcareous salts in the bones, the blood and the urine."

These quotations have been a little tedious, but they are important, and to the point. I know of no other medicinal agent so important, and yet so neglected. As all tissues are formed from cells, and as the phosphate plays an important part in the formation of cells, as well as in their subsequent changes, an abnormal diminution of it in the system must be attended with serious consequences, radical in their nature. And when there is a deficiency of the supply, what can be more natural than to increase the supply?

USES.—From what has been already said, the indications for the use of this substance may be inferred. In many cases of defective nutrition, it relieves like a charm. Relief is often so promptly gained that it is regarded as incidental. t is often objected that the texture of the bony tissues could not be so suddenly changed, as if only the bones are to be affected by the medicine. Its influence over the cell formation would lead the thoughtful to expect very prompt results.

The subphosphate may be profitably administered to children when the process of dentition is tedious and difficult. This remark will apply to second as well as to first dentition.

In well marked cases of defective nutrition, unaccompanied with positive signs of indigestion, its use will nearly always prove beneficial. And almost all American women will do themselves and their offspring a kindness by a free use of it during pregnancy and lactation. With fashionable diet, a woman can scarcely supply the waste of this salt for her own system. How, then, can she be expected to furnish a sufficient supply for a new creature, with such a wonderful growth as that from conception till the period of weaning?

In difficult and defective first dentition it is often advisable to supply both mother and child with the subphosphate, as this salt, in most cases, will be found deficient in quantity in the mother's milk. It would be easy to cite cases in confirmation of these principles, but space forbids. An extended and careful experience of more than twenty years gives me more and more confidence in their correctness.

ADMINISTRATION.—The subphosphate of lime should be regarded as a food, rather than as a medicine. Articles of food containing it abundantly should be used. Unbolted flour, cracked wheat, lean meat, fresh milk, cheese, etc., abound in it. As the pure salt is tasteless, it may be added to almost any kind of food, without in the least deteriorating any of its properties. I frequently give it to children with their bread and milk. It should be taken with the food, not on an empty stomach.

An adult may take twenty or thirty grains a day. To a babe I would give but five or six.

OBJECTIONS CONSIDERED.—1. "There is no use in giving this; for usually the fault is with the powers of assimilation."

The powers of assimilation can not assimilate any thing, unless it is present to be assimilated. They do not create. Besides, the subphosphate only needs to be taken into the system; it is already assimilated, that is, it is a chemical compound exactly as the system wants it for a chemical purpose.

2. "Its administration during pregnancy is dangerous, on account of excessive bony development of the fœtus."

This is more specious than real. The increased vitality of the mother far more than compensates. For easy child-birth, healthy women, not puny babes, are wanted. Mrs. M. used the subphosphate, by my advice, and gave birth to three boys in succession, weighing respectively 12½, 13 and 14 pounds, and had easier labors than previously with much smaller children.

3. "But this idea of 'chemical food' is a humbug."

Will the objector tell us of some kind of *food that is not chemical?* All physiological and pathological changes take place in obedience to the laws of chemistry.

Other objections, of minor importance, might be noticed; but let this much suffice.

